# Lipidomics and metabolomics investigation into the effect of DAG dietary intervention on hyperuricemia in athletes

**DOI:** 10.1016/j.jlr.2024.100605

**Published:** 2024-07-25

**Authors:** Fangyingnan Zhang, Wei Ling Florence Lim, Yuan Huang, Sin Man Lam, Yonghua Wang

**Affiliations:** 1 School of Food Science and Engineering, South China University of Technology, Guangzhou, Guangdong, China; 2 LipidALL Technologies Company Limited, Changzhou, Jiangsu Province, People's Republic of China; 3 Ersha Sports Training Center of Guangdong Province, Guangzhou, Guangdong, China; 4 State Key Laboratory of Molecular Developmental Biology, Institute of Genetics and Developmental Biology, Chinese Academy of Sciences, Beijing, People's Republic of China

**Keywords:** lipidomics, Metabolomics, elevated uric acid, plasmalogen lipids, acylcarnitines, reactive oxygen species, mitochondria

## Abstract

The occurrence of hyperuricemia (HUA; elevated serum uric acid) in athletes is relatively high despite that exercise can potentially reduce the risk of developing this condition. Although recent studies have shown the beneficial properties of DAG in improving overall metabolic profiles, a comprehensive understanding of the effect of DAG in modulating HUA in athletes is still lacking. In this study, we leveraged combinatorial lipidomics and metabolomics to investigate the effect of replacing TAG with DAG in the diet of athletes with HUA. A total of 1,074 lipids and metabolites from 94 classes were quantitated in serum from 33 athletes, who were categorized into responders and non-responders based on whether serum uric acid levels returned to healthy levels after the DAG diet intervention. Lipidomics and metabolomics analyses revealed lower levels of xanthine and uric acid in responders, accompanied by elevated plasmalogen phosphatidylcholines and diminished acylcarnitine levels. Our results highlighted the mechanisms behind how the DAG diet circumvented the risk and effects associated with high uric acid via lowered triglycerides at baseline influencing the absorption of DAG resulting in a decline in ROS and uric acid production, increased phospholipid levels associated with reduced p-Cresol metabolism potentially impacting on intestinal excretion of uric acid as well as improved ammonia recycling contributing to decreased serum uric acid levels in responders. These observed alterations might be suggestive that successful implementation of the DAG diet can potentially minimize the likelihood of a potentially vicious cycle occurring in high uric acid, elevated ROS, and impaired mitochondrial metabolism environment.

Hyperuricemia (HUA) is a metabolic condition of elevated uric acid levels in the blood, also known as purine metabolism disorder. The prevalence of HUA in Chinese adults was estimated at 17.7%–25%, with a 2-fold higher occurrence in males compared to females in China (1, 2, 3), and sexually dimorphic association with age (1). HUA is defined as serum uric acid exceeding 360 μmol/L or 6 mg/dl in females and 420 μmol/L or 7 mg/dl in males (4, 5). Under normal physiological conditions, the body removes uric acid predominantly through urination. When the body fails to efficiently remove uric acid, however, excessive levels of uric acid can form monosodium urate (MSU) crystals that accumulate over time around the joints in the form of gout (6, 7), and in the kidneys as kidney stones (1, 8, 9), resulting in inflammation and pain. Within China between 2015-2017, HUA prevalence was estimated at 185.31 million people, while gout patients were at 25.56 million people (1). In gout sufferers, MSU crystals had also been discovered inside their tendon tissues (10, 11). MSU crystals reduce tenocyte (characteristics cells in the tendons) viability through decreasing tendon collagens, thus impacting normal tendon function and increasing the likelihood of tendon injuries (10) and elevated levels of uric acids also increase the risk of developing diseases such as diabetes (12, 13), cardiovascular diseases (14, 15) and hypertension (12, 16, 17, 18). Elevated serum uric acid (SUA) levels could arise from (1) enhanced consumption of purine-rich foods, (2) increased purine catabolism by the liver, and (3) abated clearance of SUA due to renal dysfunction or a combination of these factors.

Prolonged duration of sedentary behavior (≥10 h of sitting) also increases the likelihood of HUA (19). Reduction in sedentary duration and regular physical activity decreases the probability of developing HUA. In the opposite spectrum of a sedentary lifestyle, athletes represent a group of physically active individuals. Profuse sweating was shown to increase SUA levels post-exercise and reduce the excretion of uric acid via urine (20). In a cross-sectional study on adolescent athletes, 48.9% were found to develop HUA, with the likelihood of HUA increasing with higher BMI and obesity (21). The type of physical activity also influences SUA, with sprinters demonstrated to possess lower post-exercise uric acid concentration compared to endurance runners (22). Whilst physical activity generally reduces the likelihood of HUA, conflicting findings exist that the prevalence of HUA in athletes is shown to be relatively high. A deeper understanding of the mechanisms underlying the development of HUA in athletes is henceforth necessary.

Dietary fats can exist in the forms of triacylglycerol (TAG) and diacylglycerol (DAG). The DAG used in our dietary intervention mainly comprises 1,3-DAGs that are broken down into glycerol and free fatty acids by 1,3-lipase, contrary to TAG that are hydrolyzed into 2-monoacylglycerol and free fatty acids (23). The potential advantages of incorporating DAG into diets have spurred renewed interest in recent years. In a randomized double-blinded trial, for example, alpha-linolenic acid (ALA)-DAG consumption was found to significantly increase dietary fat oxidation and reduce visceral fat deposit compared to ALA-TAG diet (24). DAG (Kao Corporation) was found to reduce fasting insulin and improve insulin resistance in normal BMI individuals (25). DAG (30% of 1,2-diacylglycerol and 70% 1,3-diacylglycerol) diet also significantly lowers postprandial TAG levels and reduces insulin secretion as compared to TAG diet (26). While dietary replacement of TAG with DAG has been shown to improve general metabolic profiles, its effectiveness in ameliorating HUA has not been investigated. Our study aims to provide insights and address the gap in this knowledge using comprehensive lipidomics and metabolomics approaches.

## Materials and Methods

### Study design and participants

This study was approved by the Ethics Committee of Guangdong Provincial Hospital of Traditional Chinese Medicine, approval number BF2020-214-01 and abided by the Declaration of Helsinki principles. Written informed consent was obtained from the study participants. This study comprised 33 athletes between the ages of 12–35 years old from Ersha Island training center in the Guangdong province. All participants enrolled in this study were professional athletes who specialized in a high-intensity competitive sport (such as badminton, volleyball, martial arts, boxing, fencing, and synchronised swimming) with competition commitments and comparable amounts of exercise. These athletes had HUA at baseline and were subjected to DAG (soy glycerol diglycerides; 80% soybean diester containing 75% 1,3-diacylglycerol and 25% 1,2 diacylglycerol/2,3-diacylglycerol and 20% soybean triester) diet intervention for a two-month period. The central canteen at the training center used DAG oil which was provided by Guangzhou Yong Hua Special Nutrition Technology Co., Ltd to prepare and provide centralized meals at a frequency of 3 meals each day for the study participants. Athletes who participated in other clinical studies within 30 days prior to the screening period were excluded. At the end of the DAG diet intervention, blood samples for lipidomics and metabolomics analyses were collected from the athletes after overnight fasting (T1). Clinical indices were collected at both baseline (T0) and after DAG diet intervention (T1). Body mass index (BMI) was calculated as weight/height^2^. Table 1 summarizes the clinical characteristics of the recruited athletes in this study. These HUA athletes were categorized into two groups namely: responders and non-responders. Responders referred to HUA athletes whose uric acid levels returned to normal levels after DAG diet intervention (<420 μmol/L for males, <360 μmol/L for females), while non-responders referred to HUA athletes who still possessed higher than normal blood uric acid levels after DAG diet intervention.

**Table 1:** Characteristics of study participants

Groups	All			Males			Females		
	Responders N = 12^a^	Non-responders, N = 21^a^	*P*-value	Responders, N = 8^a^	Non-responders, N = 15^a^	*P*-value	Responders, N = 4^a^	Non-responders, N = 6^a^	*P*-value
				<420	≥420		<360	≥360	
Age	17.8 (1.9)	18.9 (2.2)	0.2^b^	18.0 (1.8)	19.6 (1.9)	0.062^b^	17.5 (2.4)	17.0 (1.7)	0.7^b^
T0 BMI	21.9 (3.4)	21.8 (3.7)	>0.9^b^	23.0 (3.4)	23.1 (3.4)	>0.9^b^	19.7 (2.4)	18.9 (3.1)	0.7^b^
T1 BMI	21.7 (3.1)	22.5 (3.0)	0.5^b^	22.7 (2.9)	23.2 (3.1)	0.7^b^	19.5 (2.5)	20.3 (1.6)	0.6^b^
T0 Uric acid, μmol/L	491.0 (74.9)	510.3 (78.1)	0.5^b^	523.3 (56.3)	504.2 (86.6)	0.5^b^	426.5 (69.8)	525.5 (54.9)	0.059^b^
T1 Uric acid, μmol/L	345.1^c^ (41.3)	462.3^c^ (51.3)	<0.001^b^	365.0^c^ (25.4)	469.3 (40.7)	<0.001^b^	305.3^c^ (39.7)	444.8 (73.3)	0.005^b^
Dining_days	46.4 (14.3)	44.8 (12.4)	0.7^b^	42.7 (14.4)	42.3 (13.3)	>0.9^b^	53.9 (12.5)	51.1 (7.2)	0.7^b^
T0 Aspartate aminotransferase, U/L	27.3 (9.9)	23.7 (7.6)	0.3^b^	31.0 (9.9)	23.9 (7.0)	0.10^b^	20.0 (4.3)	23.0 (9.7)	0.5^b^
T1 Aspartate aminotransferase, U/L	24.8 (13.8)	22.6 (6.6)	0.6^b^	28.3 (16.0)	23.1 (5.7)	0.4^b^	18.0 (3.8)	21.3 (8.9)	0.4^b^
T0 Alanine aminotransferase, U/L	18.6 (7.5)	16.7 (8.4)	0.5^b^	17.8 (5.6)	18.1 (9.1)	>0.9^b^	20.3 (11.4)	13.0 (5.1)	0.3^b^
T1 Alanine aminotransferase, U/L	15.7 (9.2)	16.6 (6.6)	0.8^b^	16.6 (9.5)	18.7 (6.4)	0.6^b^	13.8 (9.6)	11.2 (2.9)	0.6^b^
T0 Triglycerides, mmol/L	0.6 (0.2)	1.0 (0.5)	0.007^b^	0.7 (0.2)	1.0 (0.5)	0.049^b^	0.6 (0.2)	0.9 (0.3)	0.050^b^
T1 Triglycerides, mmol/L	0.9^c^ (0.4)	1.0 (0.4)	0.4^b^	1.0 (0.4)	1.0 (0.4)	0.8^b^	0.9 (0.4)	1.1 (0.2)	0.2^b^
T0 Blood glucose, mmol/L	4.3 (0.3)	4.4 (0.4)	0.5^b^	4.4 (0.3)	4.4 (0.3)	>0.9^b^	4.1 (0.3)	4.4 (0.5)	0.3^b^
T1 Blood glucose, mmol/L	4.5 (0.3)	4.6 (0.3)	0.5^b^	4.6 (0.3)	4.5 (0.3)	0.5^b^	4.3 (0.2)	4.8 (0.3)	0.024^b^
T0 Fasting insulin, μU/ml	5.2 (2.9)	8.4 (6.3)	0.061^b^	5.5 (3.3)	8.7 (7.2)	0.2^b^	4.7 (2.0)	7.4 (3.9)	0.2^b^
T1 Fasting insulin, μU/ml	7.1 (3.5)	9.3 (4.2)	0.11^b^	5.6 (2.4)	8.3 (3.7)	0.046^b^	10.0 (3.7)	11.9 (4.6)	0.5^b^

*P*-values were calculated using Welch two sample *t* test and Fisher’s exact test for numerical and categorical variables, respectively. Comparison was also performed for individual clinical variables at T0 (baseline) and T1 (after DAG diet intervention), clinical variables with *P*-values < 0.05 between T0 and T1 were marked with ∗.

a Mean (SD); n/N (%).

b Welch Two Sample *t* test.

c Denoted *P*-values <0.05 between T0 and T1.

### Lipidomic analyses

Lipids were extracted from 50 μl of serum using a modified version of Bligh and Dyer’s method (27). Briefly, 750 μl of chloroform: methanol (1:2, v/v) was added to serum samples and incubated at 1,500 rpm for 30 min at 4°C. After incubation, 350 μl of deionized water and 250 μl of chloroform were added to induce phase separation. The samples were then centrifuged and the lower organic phase containing lipids was extracted into a clean tube. Lipid extraction was repeated once by adding 450 μl of chloroform to the remaining aqueous phase and the lipid extracts from both rounds were pooled into a single tube and dried in the SpeedVac under OH mode. The dried samples were stored at −80°C until further analysis and reconstituted in 300 μl of chloroform: methanol: ammonium acetate (100:100: 4; v/v/v) prior to lipidomic analyses. Lipidomic analyses were conducted at LipidALL Technologies using a Jasper HPLC coupled with Sciex Triple Quad 4,500 MD (28). Separation of individual lipid classes was performed with normal phase (NP) high-performance liquid chromatography (HPLC) using a TUP-HB silica column (i.d. 150 × 2.1 mm, 3 μm) with the following conditions: mobile phase A (chloroform: methanol: ammonium hydroxide, 89.5:10:0.5) and mobile phase B (chloroform: methanol: ammonium hydroxide: water, 55:39:0.5:5.5). Multiple reaction monitoring (MRM) transitions were set up for comparative analysis of various lipids. Individual lipid species were quantified by referencing spiked internal standards. Internal standards d9-PC32:0 (16:0/16:0), PE 34:0, diC8-PI, d31-PS, C17:0-PA, DMPG, CL-14:0, C14-bis (monoacylglycerol)phosphate (BMP), C12-sulfatide (SL), C17-LPC, C17-LPE, C17:1-LPI, C17:0-LPA, C17:1-LPS, C17-Cer, C12-SM, d17:1-sphingosine-1-phosphate (S1P), d17:1-sphingosine (Sph), C8-glucosylceramide (GluCer), C8-lactosylceramide (LacCer), globotriaosylceramides (Gb3)-d18:1/17:0, d3-16:0 carnitine, DAG(18:1/18:1)-d5 were obtained from Avanti Polar Lipids. Internal standard monosialodihexosyl ganglioside (GM3)-d18:1/18:0-d3 was purchased from Matreya LLC. Free fatty acids were quantitated using d31-16:0 (Sigma-Aldrich). Internal standards d6-CE 18:0 and TAG (16:0)3-d5 were obtained from CDN isotopes.

### Metabolomics analyses

Untargeted metabolomics was conducted at LipidALL Technologies. Polar metabolites were extracted from 100 μl of serum using 400 μl of ice-cold methanol containing 0.28 mM phenylhydrazine. Samples were vortexed and kept at −20°C for 1 h for derivatization of alpha-keto acids (29). Following derivatization, the samples were centrifuged at 12,000 rpm for 10 min at 4°C. Clean supernatant was transferred to a new tube and dried in a SpeedVac under H_2_O mode. The dried extract was reconstituted in 5% acetonitrile in water prior to LC-MS analysis on an Agilent 1,290 II UPLC coupled to Sciex 5,600+ quadrupole-TOF MS. Polar metabolites were separated on a reverse-phased Waters ACQUITY HSS-T3 column (3.0 × 100 mm, 1.8 μm). MS parameters for detection were: ESI source voltage positive ion mode 5.5 kV, negative ion mode −4.5 kV; vaporizer temperature, 500°C; drying gas (N2) pressure, 50 psi; nebulizer gas (N2) pressure, 50 psi; curtain gas (N2) pressure, 35 psi: the scan range was m/z 60–800 (30). Information-dependent acquisition mode was used for MS/MS analyses of the metabolites. Collision energy was set at (±) 35 ± 15 eV. Data acquisition and processing were performed using Analyst® TF 1.7.1 Software (AB Sciex, Canada). All detected ions were extracted using MarkerView 1.3 (AB Sciex) into Excel in the format of a two-dimensional matrix, including mass-to-charge ratio (m/z), retention time, and peak areas, and isotopic peaks were filtered. PeakView 2.2 (AB Sciex) was applied to extract MS/MS data and perform comparisons with the Metabolites database (AB Sciex), HMDB, and standard references to annotate ion identities (31). A cocktail of isotopically-labelled internal standards (IS) purchased from Cambridge Isotope Laboratories were spiked into the samples for metabolite quantitation, including Guanosine (13C10; 15N5), Thymidine (13C10; 15N2), L-lactate-D3, Kynurenic acid-D5, Fumaric acid-D2, Malic acid-D3, L-Aspartic acid-D3, Succinic acid-D4, Glutaric acid-D4, Methylsuccinic acid-D6, Citric acid-D4, Benzoic acid-D5, P-cresol sulfate-D7, Hippuric acid-D5, Pyruvate-d3, Oleic acid-d9, Urea (13C; 15N2), Glycine-13C2, Trimethylamine N-oxide-D9, Sarcosine-D3, L-Alanine-D4, L-Serine-D3, Creatinine-D3, Choline-D13, L-Proline-D7, L-Threonine (13C4; 15N), L-Valine-D8, Taurine-13C2, Betaine-D11, 4-Hydroxyproline-D3, Creatine-D3, Hypoxanthine-D3, L-Isoleucine-D10, L-Leucine-D10, L-Glutamine-D5, L-Methionine-D3, L-Glutamate-D5, Xanthine-15N2, L-Lysine-D9, L-Histidine-13C6, L-Carnitine trimethyl-d9, Uric acid (13C; 15N3), L-Citrulline-D4, L-Arginine-D7, L-Tyrosine-D7, L-Tryptophan-D5, Uridine-D2, Cytidine-13C5, Inosine-15N4, Carnitine-C12:0-d9, Carnitine-C14:0-d9, Carnitine-C16:0-d3, Glycodeoxycholate-d4. Peak areas of endogenous metabolites were normalized to the areas of their corresponding isotopically labeled structural analogs for quantitation. For endogenous metabolites without labeled structural analogs, an automated algorithm selects the optimal internal standard for quantitation based on the rule of minimal coefficients of variations (COVs) after normalization.

### Statistical analysis

All data analyses were performed in R version 4.2.3. Principal Component Analysis (PCA) was used to visualize the clustering of quality control (QC) samples. Characteristics of study participants were compared using Welch's two-sample *t* test and Fisher’s exact test for numerical and categorical variables, respectively. A pairwise scatter plot of log10-transformed concentration of individual metabolite species was used to visualize the consistency between the QC samples. A lipid change network was created to visualize the overall differences among lipids from various classes using *P* values from Limma analysis and log2 fold-changes in responders relative to non-responders. The size of the circle corresponded to the magnitude of *P* value. Increases were indicated in red and decreases were indicated in blue. A heatmap was used to visualize the overall difference in metabolomes for all the samples. Increases were indicated in red and decreases were indicated in blue. The intensity of the color denoted the number of standard deviations from the average. Differential analysis was performed using the Limma R package (32), accounting for age, gender, baseline uric acid and dining days using log2-transformed lipidome and metabolome data. *P*-values were corrected with the Benjamini-Hochberg method. Log2 fold-change and *P*-values from the Limma model were illustrated as volcano plots that compare changes in responders versus non-responders. Statistically significant lipids/metabolites that were increased in responders relative to non-responders were denoted as red, while lipid/metabolites that were decreased were in blue. Non-significant lipids/metabolites were in grey. To investigate the changes in coregulation amongst lipids, metabolites, and clinical indices between non-responders and responders, differential correlation analysis was performed using the R package “differential gene correlation analysis” (DGCA) (33). DGCA identifies a pair of variables with significantly different Spearman correlations and classifies these patterns into subgroups such as −/+, 0/+, +/++, etc. A negative correlation was denoted as − and a positive correlation was denoted as +; −/+ denotes that the correlation was negative in non-responders but positive in responders. A network was constructed from the differential correlated pairs (*P* < 0.05) and further analyzed using the R package “Multiscale Embedded Gene Co-Expression Network Analysis” (MEGENA) (34). Gene set enrichment analysis (GSEA) was performed using “clusterProfiler” (35) R package with the small molecular pathway database (SMPDB) (36) from “MetaboAnalystR” (37) R package and based on metabolites with t-statistic from the Limma analyses controlling for confounders (age, gender, dining days and baseline uric acid).

## Results

### Omics analyses and clinical characteristics of study participants

**Fig. 1:**
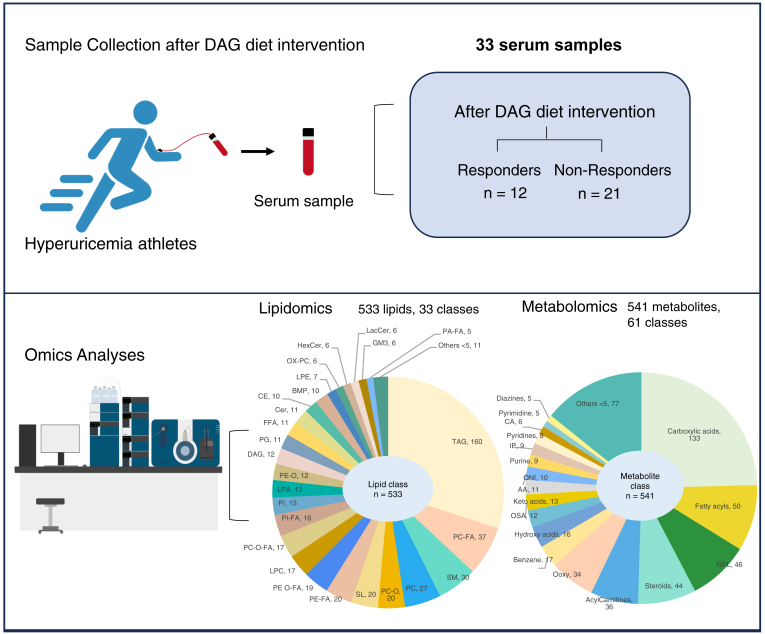
Study design and analysis approaches. Serum samples were collected from 33 athletes via venipuncture after DAG diet intervention and the study participants were separated into two groups (responders and non-responders). There were 12 responders and 21 non-responders. A total of 1,074 metabolites (including 533 lipids from 33 classes and 541 polar metabolites from 61 classes) were analyzed using lipidomics and metabolomics approaches. AA, amino acids; BMP, Bis[monoacylglycero]phosphates; CA, cinnamic acids; CE, cholesterol ester; CER, ceramides; DAG, diacylglycerol; FFA, free fatty acyl; GM3, monosialodihexosylganglioside; GPL, glycerophospholipids; HexCer, hexosylceramides; IP, imodazopyrimidines; LacCer, lactosylceramide; LPC, lysophosphatidylcholine; LPA, lysophosphatidic acid; LPE, lysophosphatidylethanolamine; OX-PC, oxidized phosphatidylcholine; Ooxy, organooxygen; OSA, organic sulfuric acids; ONI, organonitrogen; PA-FA, phosphatidic acid-formamides; PC, phosphatidylcholine; PC O, plasmalogen phosphatidylcholine; PC O-FA, plasmalogen phosphatidylcholine-formamides; PC-FA, phosphatidylcholine-formamides; PE-FA, phosphatidylethanolamine-formamides; PI-FA, phosphatidylinositol-formamides; PI, phosphatidylinositol; PE-O, plasmalogen phosphatidylethanolamine; PG, phosphatidylglycerol; SL, sulfatides; SM, sphingomyelin; TAG, triacylglycerol.

Serum samples were collected from 33 athletes via venipuncture after an overnight fast at the end of the DAG diet intervention period. A total of 1,074 metabolites (including 533 lipids from 33 classes and 541 polar metabolites from 61 classes) were quantitated using lipidomics and metabolomics approaches (Fig. 1). Quality control (QC) samples were prepared from aliquots of pooled biological samples and hence represented the averaged lipidome and metabolome profiles across all samples analyzed. The QC samples were inserted between every ten biological samples across the MS runs. Principal component analysis (PCA) and correlation plots of QC samples were centered and clustered together, and the QC profiles were highly correlated confirming the reproducibility of our LC-MS data (supplemental Fig. S1A, B).

The clinical characteristics of the study participants are shown in Table 1. The study participants were athletes with HUA at baseline, who were categorized into two groups based on serum uric acid levels at the end of DAG diet intervention, namely responders (n = 12) (females <360 μmol/L, males <420 μmol/L) and non-responders (females ≥ 360 μmol/L, males ≥ 420 μmol/L) based on cutoff values determined from previous studies (3, 4, 5, 38). All participants maintained healthy BMI both at baseline and after the DAG diet intervention (Table 1). In accordance with a previous study (1), we also observed a higher prevalence of HUA in males (Table 1). Serum uric acid after DAG diet intervention was decreased in both responders and non-responders, with a steeper decline observed for responders regardless of gender (Table 1). Responders exhibited significantly lower serum uric acid levels after DAG diet intervention than non-responders, despite comparable levels of serum uric acid at baseline. It was also notable that responders displayed appreciably lower serum triglycerides than non-responders at baseline, and the differences in serum triglycerides between responders and non-responders disappeared at the end of the DAG diet intervention. Blood glucose was lower in female responders than non-responders at the end of the intervention, while no notable changes in fasting insulin were observed. Taken together, DAG diet intervention was generally effective in reducing serum uric acid levels, but with notable differences in terms of effectiveness between responders and non-responders.

### Changes in serum lipidome after DAG diet intervention

**Fig. 2:**
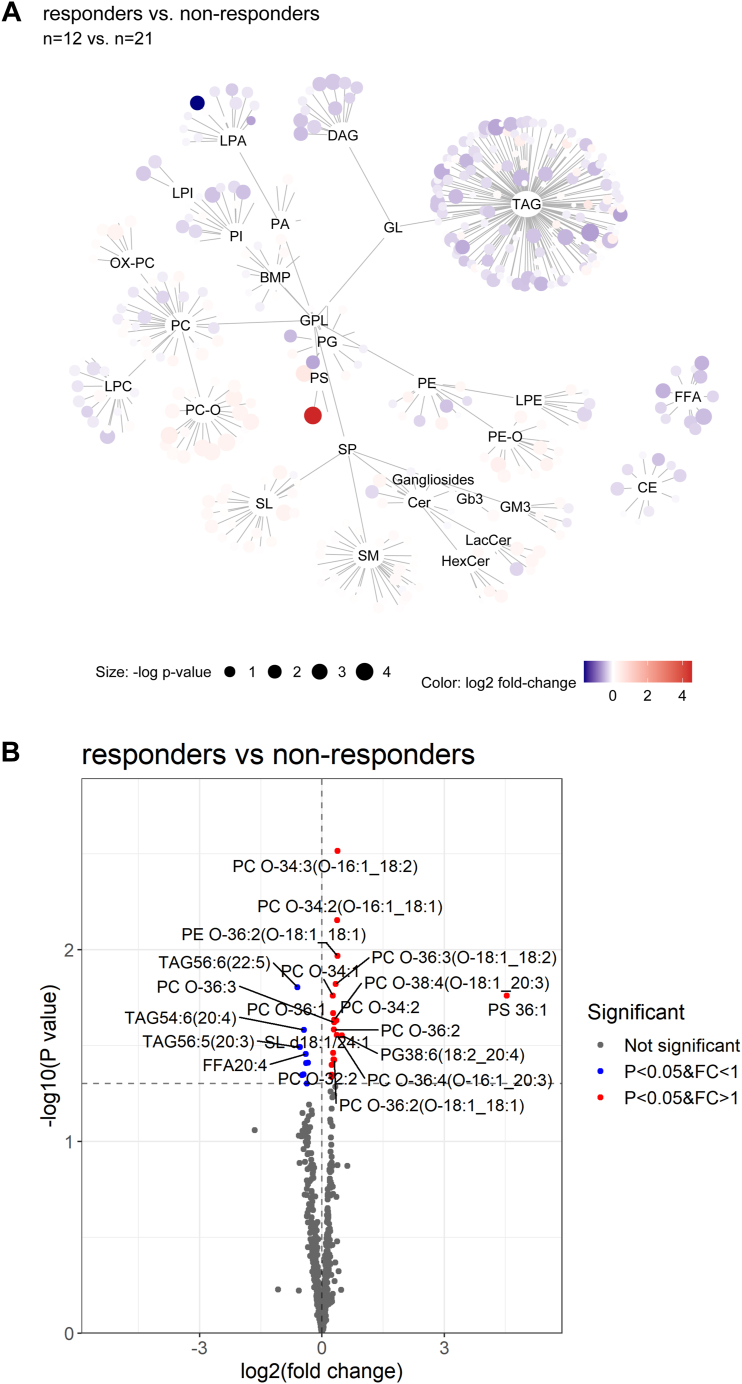
Lipids associated with responders. A: lipid change network was created to visualize the overall differences among the lipid classes using *P* value of Limma analysis and log2 fold-change. Size of the circle corresponded to the *P* value. Positive log2 fold-changes were indicated in red and negative log2 fold-changes were indicated in blue. Plasmalogen lipids (PC O and PE O) were negatively associated and TAG and DAG were positively associated. B: Volcano plot of the top 20 most statistically significant lipid changes associated with responders. Positively significant lipids with greater than 1-fold change were denoted as red, negatively significant lipids with less than 1-fold change were denoted as blue and the non-significant lipids were denoted as grey. *P* values (*P* < 0.05) were determined by limma analysis.

To interrogate serum lipid changes associated with the effectiveness of serum uric acid reduction, a linear model controlling for age, gender, baseline uric acid levels and the number of dining days was constructed using the Limma package in R. Lipid change network was used to visualize the global serum lipid differences between responders and non-responders after DAG diet intervention (Fig. 2A). A complete list of statistically significant lipids was listed in supplemental Table S1. Profound changes were observed in the levels of plasmalogen phosphatidylcholines (PC Os) and TAGs in responders relative to non-responders (Fig. 2A). A volcano plot illustrating the top 20 most statistically significant lipids ranked by *P*-values showed that majority of these top altered lipids were PC Os and TAGs (Fig. 2B). Plasmalogen lipids including PC O 34:3 (O-16:1_18:2) and PC O 34:2 (O-16:1_18:1) were elevated in responders, while polyunsaturated neutral lipids such as TAG56:6 (22:5), TAG54:6 (20:4), TAG56:5 (20:3) and FFA 20:4 were significantly decreased (Fig. 2B). The top 20 most significant altered lipids between responders and non-responders were also shown as boxplots (supplemental Fig. S2). Among the top 20 altered lipids, 13 of these lipids were plasmalogen lipids that were consistently elevated in the serum of responders as compared to non-responders (supplemental Fig. S2). Taken together, in responders there were significant increases in plasmalogen lipids especially PC Os, while circulating TAGs were significantly decreased compared to non-responders.

### Changes in serum metabolome after DAG diet intervention

**Fig. 3:**
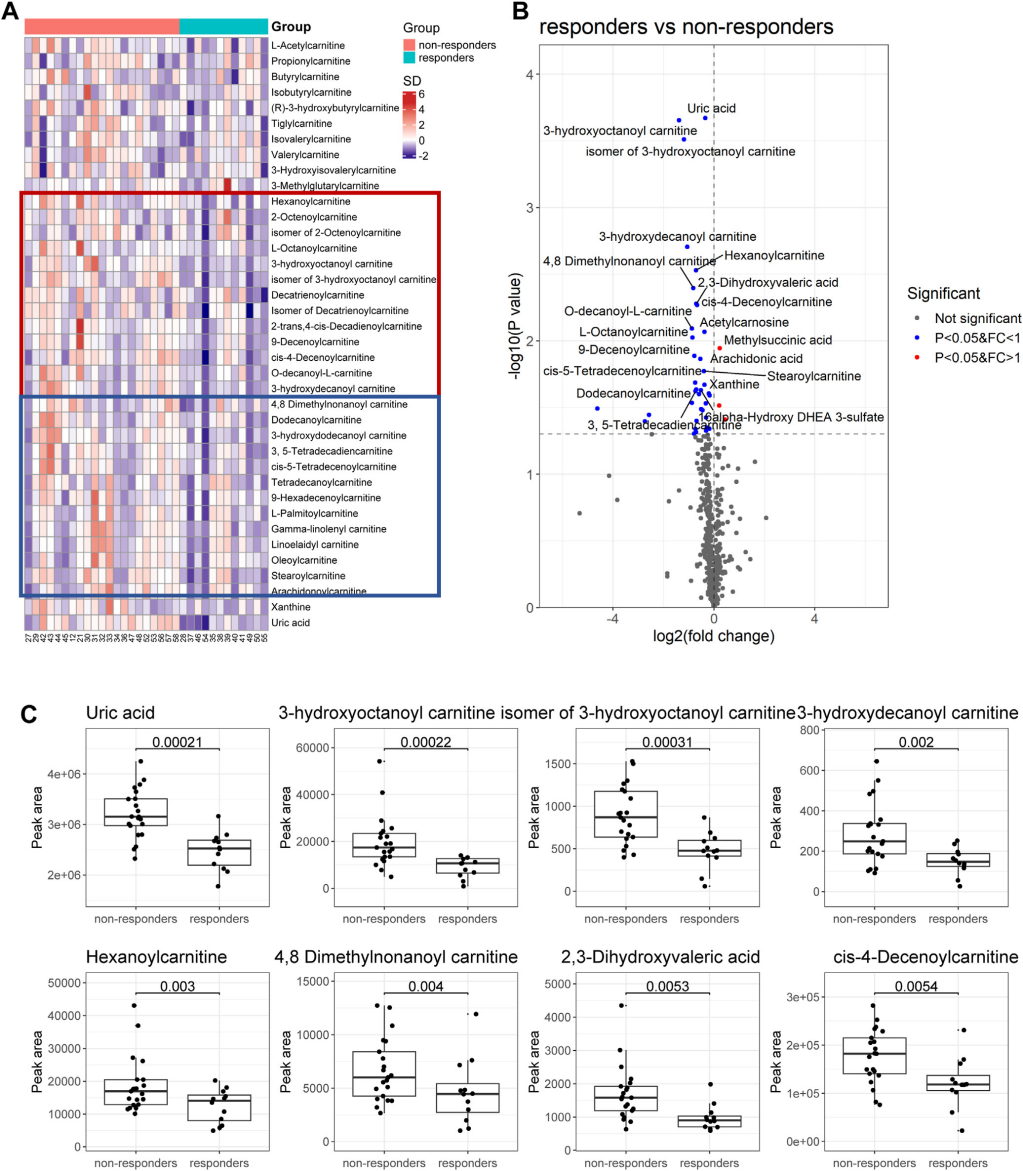
Metabolites associated with responders. A: Heatmap of metabolites (acylcarnitines, xanthine, and uric acid) between responders and non-responders. The color of the heatmap indicated the magnitude of the SD. For grouping, the pink band denoted non-responders and cyan band denoted responders. B: Volcano plot of the top 20 most statistically significant metabolite changes associated with responders. Positively significant metabolites with greater than 1-fold change were denoted as red, negatively significant metabolites with less than 1-fold change were denoted as blue and the non-significant metabolites were denoted as gray. *P* values (*P* < 0.05) were determined by limma analysis. C: Boxplot of the top 8 statistically significant metabolites. FC, fold change; SD, standard deviation.

A linear model controlling for age, gender, baseline uric acid levels, and the number of dining days based on metabolome data revealed a predominance of changes in serum acylcarnitines (Fig. 3). Acylcarnitines are classified into different subclasses based on acyl chain carbon atom numbers (C), including short-chain acylcarnitines (C2 -5), medium-chain acylcarnitines (C6 -12), long-chain acylcarnitines (C13 to 20) and very-long-chain acylcarnitines (>C21) (39). Top significantly altered metabolites were presented in a heatmap (Fig. 3A), and a complete list of statistically significant metabolites was presented in supplemental Table S2. Medium-chain acylcarnitines (boxed red, Fig. 3A), long-chain acylcarnitines (boxed blue, Fig. 3A), and purine derivatives including xanthine and uric acid were significantly reduced in the serum of responders relative to non-responders. Medium-chain acylcarnitines (3-hydroxyoctanoyl carnitine, isomer of 3-hydroxyoctanoyl carnitine, 3-hydroxydecanoyl carnitine, Hexanoyl carnitine, cis-4-Decenoylcarnitine, O-decanoyl-L-carnitine, L-Octanoylcarnitine, 9-Decenoylcarnitine and Dodecanoylcarnitine), long-chain acylcarnitines (4,8 Dimethylnonanoylcarnitine, Stearoylcarnitines, cis-5-Tetradecenoylcarnitines and 3,5-Tetradecadiencarnitine), and purine metabolism pathway metabolites such as xanthine and uric acid were significantly decreased in responders, while methylsuccinic acid was increased. (Fig. 3B, C and Supplemental Fig. S3).

### Changes in metabolic co-regulation between responders and non-responders

**Fig. 4:**
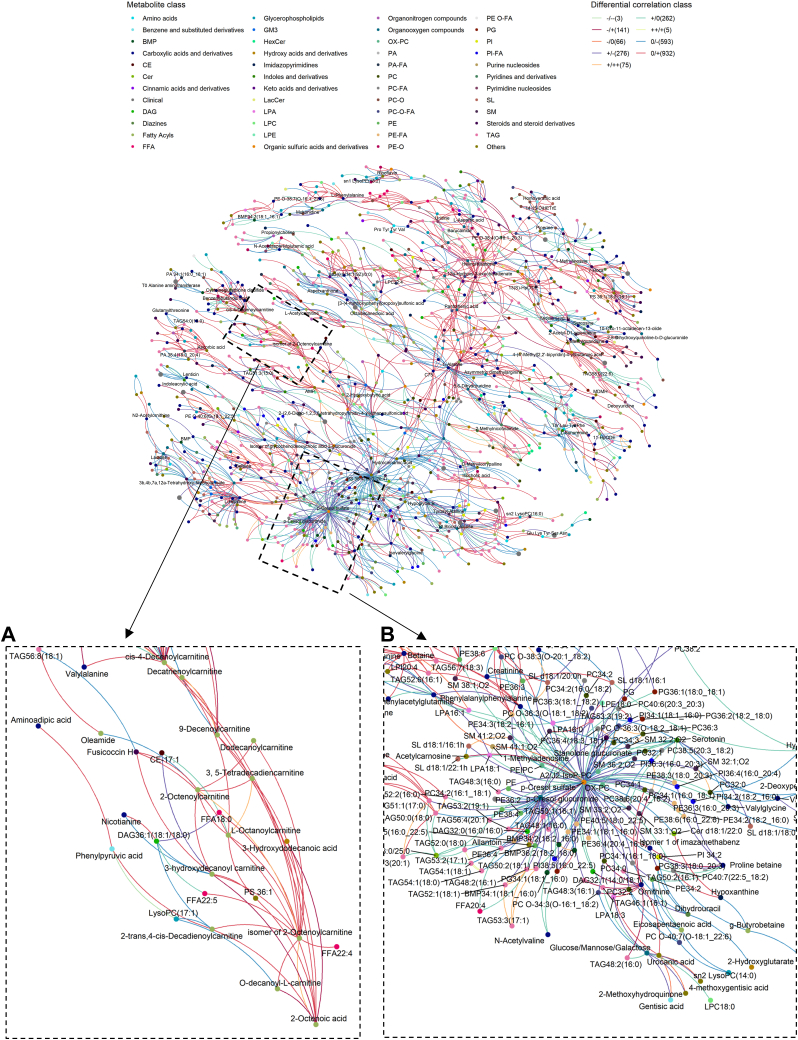
Metabolic co-regulation network using lipids, metabolites, and clinical indices. Differential gene correlation analysis (DGCA) was used to visualize differential correlation networks using the R package and differential correlated pair (*P* < 0.05) and analyzed using the R package “Multiscale Embedded Gene Co-Expression Network Analysis” (MEGENA). Correlation was calculated using Spearman correlation (A) Module consisting of acylcarnitines with 2-Octenoic acid as a central hub and (B) Module consisting of many species of glycerophospholipids including plasmalogen lipids, TAGs, and metabolites with 2 central hubs of p-Cresol sulfate and p-Cresol glucuronide. Negative correlation was denoted as - and positive correlation was denoted as +.

We utilized DGCA to investigate the changes in metabolic co-regulation between responders and non-responders (Fig. 4). A global network was constructed from the differential correlated pairs (*P* < 0.05) and further analyzed using R package “Multiscale Embedded Gene Co-Expression Network Analysis” (MEGENA). Metabolic coregulation analysis revealed two metabolite modules of outstanding interest (Modules A and B, Fig. 4). Module A comprises several acylcarnitines connected to central hub 2-Octenoic acid (medium chain fatty acid) via maroon lines (−/+), indicating that serum acylcarnitines were negatively correlated with the levels of fatty acid precursors in non-responders, and the coregulation was positive in responders. These observations imply a positive conversion of serum fatty acid precursors into acylcarnitines that were subsequently expended in responders but were accumulated in the serum of non-responders instead, thereby resulting in the negative coregulation possibly indicative of perturbed mitochondrial β-oxidation. Module B consisted of the central hubs p-Cresol sulfate and p-Cresol glucuronide connected to several glycerophospholipids, including PC Os and PEs via purple lines (+/−). The metabolic coregulation indicates that higher levels of circulating phospholipids in responders were associated with reduced amounts of p-Cresol metabolism products. Sulfatation and glucuronidation of p-Cresol occur in the liver and colonic mucosa, and its metabolism products were reported to be pro-inflammatory and predictive of mortality in patients with chronic kidney disease (40). Differential correlation analyses indicate that higher levels of circulating phospholipids such as PC Os in responders were associated with potentially lower levels of p-Cresol, and hence attenuated levels of its metabolism products. Indeed, p-Cresol, produced by intestinal bacterial fermentation, was shown to lower uric acid transport across intestinal epithelia (41), which might have consequences on the enteric excretion of uric acid that accounts for approximately one-third of total systemic uric acid excretion (42).

### Association of lipids and metabolites with clinical indices

**Fig. 5:**
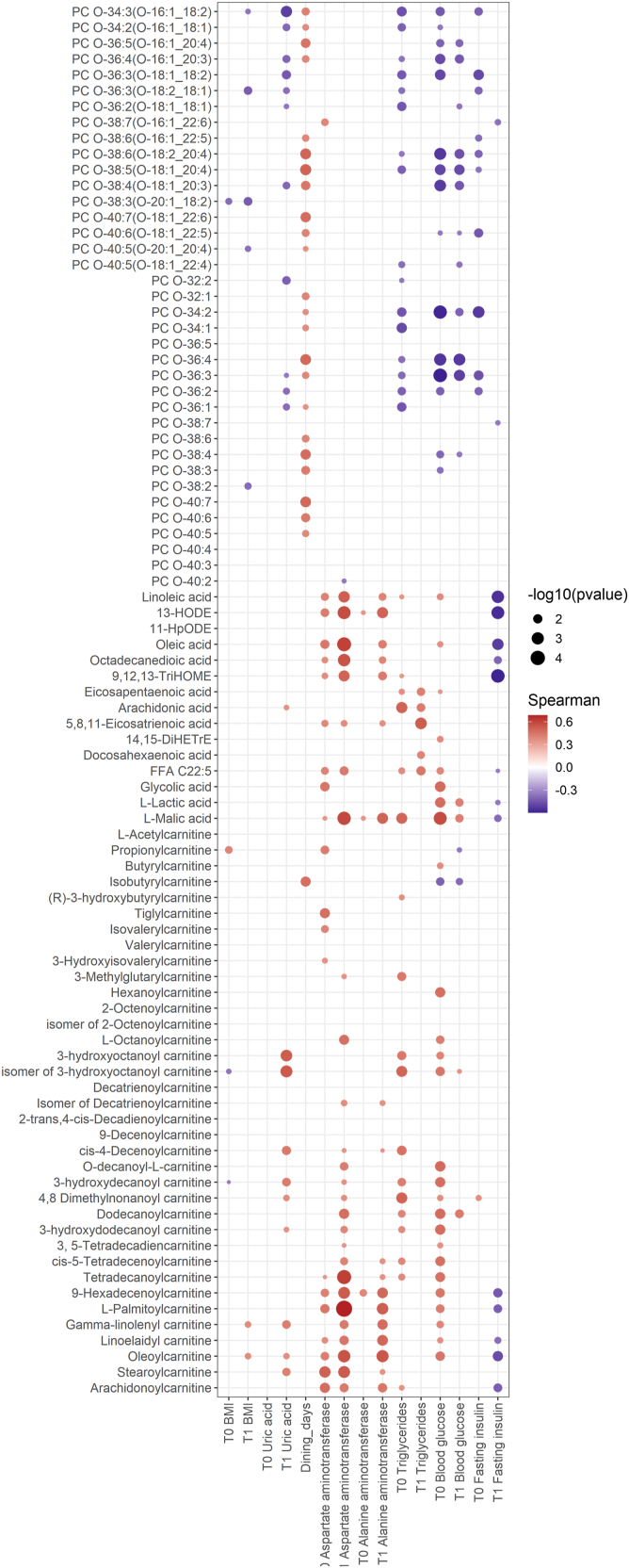
Lipids and Metabolites associated with clinical characteristics. Correlation was calculated using Spearman correlation and *P*-value <0.05 were displayed as dots. Red denoted positive correlation and blue denoted negative correlation. The size of the dots indicated the magnitude of the -log10 (*P*-value). BMI, body mass index; T0, baseline; T1, after DAG diet intervention.

Spearman correlation analysis was leveraged to examine the associations between clinical indices with lipids and metabolites differentially expressed between responders and non-responders. We emphasized the clinical associations of serum PC Os and various acylcarnitines, based on the foregoing results that identified these metabolites as key determinants of the effectiveness of DAG diet intervention in lowering serum uric acid levels. Interestingly, numerous serum PC Os were positively correlated with the number of dining days (Fig. 5), and negatively correlated with serum uric acid levels at the end of the DAG diet intervention. These observations support the notion that DAG diet intervention elicits systemic increases in PC Os that were associated with a reduction of serum uric acid levels. The number of dining days was not correlated with serum DAGs nor TAGs (supplemental Fig. S4), implying that DAGs consumed were likely converted to other forms of lipids to evoke biological effects on serum uric acid levels. PC Os were also negatively associated with blood glucose and baseline triglyceride levels (Fig. 5), aligned with observations that responders generally exhibited a better metabolic profile (ie lower blood triglycerides and glucose) at baseline. Several serum acylcarnitines were positively correlated with both serum aspartate aminotransferase (AST) and alanine aminotransferase (ALT) levels (Fig. 5), suggesting that elevated circulating acylcarnitine levels in non-responders might be associated with liver function.

### Metabolic pathways altered in responders relative to non-responders of DAG diet intervention

**Fig. 6:**
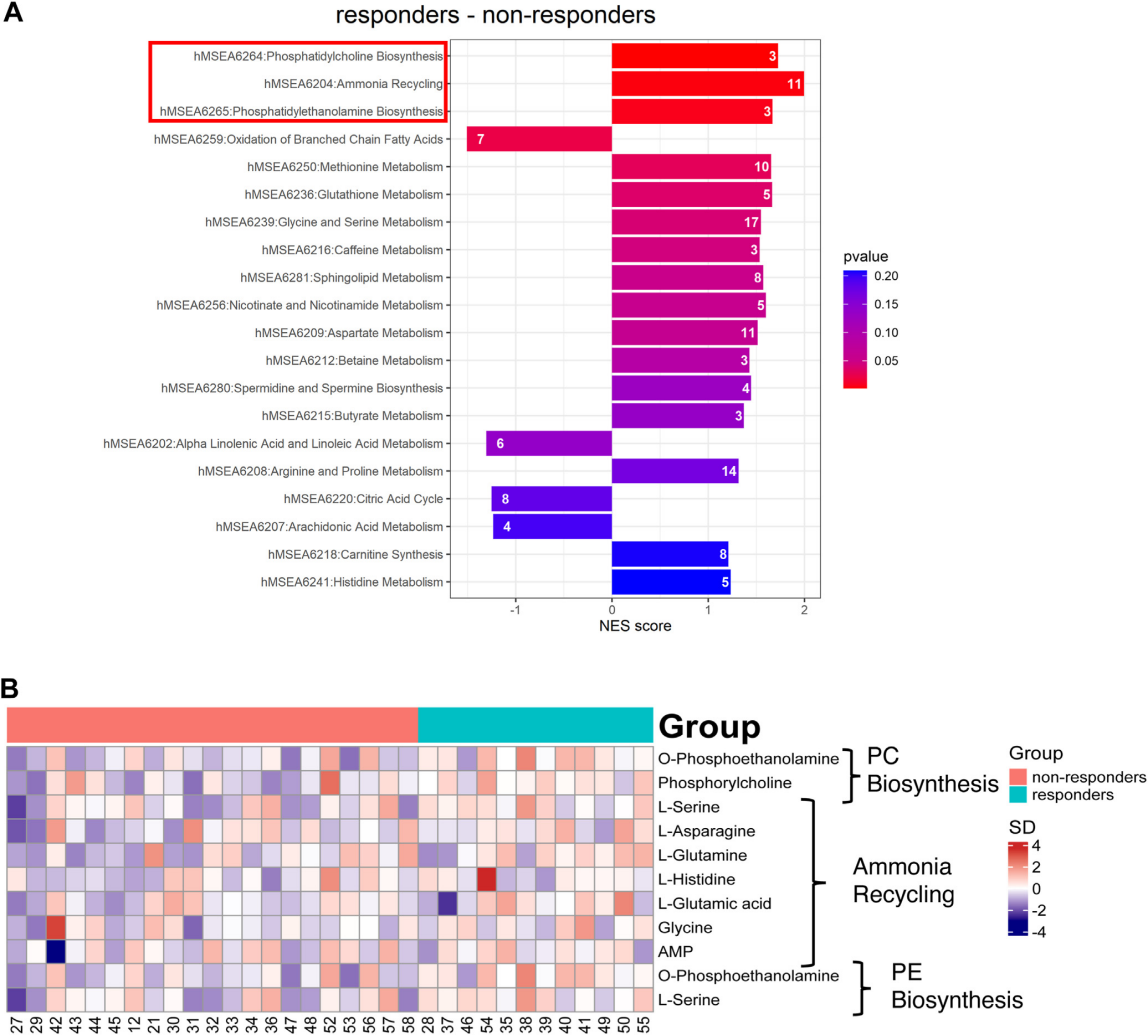
Pathways associated with responders. A: Gene set enrichment analysis (GSEA) was performed based on significant metabolites (t-statistics) from the Limma analyses controlling for confounders (age, gender, dining days, and baseline uric acid). The color of the Bar plots indicated the magnitude of the *P*-value and the number indicated in the bars denoted the number of counts. The pathways of interest are highlighted with a red rectangle. B: The metabolites involved in phosphatidylcholine biosynthesis, ammonia recycling, and phosphatidylethanolamine biosynthesis pathways were depicted as heatmaps. The color of the heatmap indicated the magnitude of the SD. NES, normalized enrichment score; PC, phosphatidylcholine; PE, phosphatidylethanolamine; SD, standard deviation.

Based on t-statistic from Limma analysis of polar metabolites, we performed gene set enrichment analysis (GSEA) using the small molecular pathway database (SMPDB) to elucidate molecular pathways significantly altered in responders relative to non-responders of DAG diet intervention (Fig. 6A). Corroborating our lipidomic findings, GSEA analyses revealed that pathways related to phospholipid metabolism, such as phosphatidylcholine biosynthesis and phosphatidylethanolamine biosynthesis, were upregulated in responders relative to non-responders. Furthermore, the molecular pathway of ammonia recycling was also enhanced in responders, with observed increases in amino acids including L-Glutamine, L-Asparagine, and glycine that participate in renal ammonia excretion compared to non-responders (Fig. 6B).

## Discussion

Under HUA, increased levels of uric acid accumulate in joints, kidneys, and tendons as MSU crystals. In the case of HUA athletes, the increased occurrence of MSU crystals in the tendons predisposes these high-risk athletes to possible tendon injuries such as Achilles tendon rupture (43). In an attempt to reduce the likelihood of sports injuries arising from HUA, DAG diet intervention was conducted to investigate the potential benefits of DAG in reducing serum uric acid in athletes, and its associated mechanisms were interrogated using lipidomics and metabolomics approaches, which identified lipidome and metabolome signatures altered in responders relative to non-responders of DAG diet intervention. Importantly, the differing effectiveness of the response to diet intervention was biological in nature, given that the number of dining days was not significantly different between responders and non-responders (Table 1). We then sought to interrogate metabolic pathways underlying the distinct biological responses to DAG diet intervention using integrated omics approaches.

Combinatorial lipidomics and metabolomics uncovered stark increases in systemic phospholipids, particularly PC Os, with concomitant reductions in acylcarnitines and purine metabolites in responders relative to non-responders. Purine metabolism pathway begins from ribose-5-phosphate and adenine triphosphate (ATP), which leads to the production of hypoxanthine that is further metabolized by xanthine oxidase (XO) or xanthine dehydrogenase (XDH) to produce xanthine and subsequently uric acid, the end-product of purine metabolism in humans (44) (supplemental Fig. S5). Both xanthine and uric acid were significantly reduced in responders to the DAG diet intervention as compared to non-responders (Fig. 3B, C and supplemental Fig. S3). Oxidation of xanthine by XO generates uric acid and reactive oxygen species (ROS), and ROS preferentially attacks vinyl ether linkages of plasmalogens (45). As such, the attenuated generation of ROS that accompanied lowered uric acid production in responders might account for the elevated circulating PC Os in this group. Aside from companion ROS generation, uric acid per se can act as an effective oxidant. There have been controversies surrounding whether uric acid is an antioxidant or an oxidant (46), which was later shown to depend on the nature of the surrounding micromilieu. Under hydrophilic conditions, uric acid scavenges ROS and acts as an antioxidant (47, 48), while in hydrophobic environments such as within lipid membranes, uric acid chiefly functions as a pro-oxidant (49, 50), particularly in the presence of oxidized lipids (50, 51). Intracellularly, soluble uric acid was shown to induce ROS production and act as a pro-oxidant (52, 53), for example, in lipid-laden human primary adipocytes (46). Hence, lowered PC Os in non-responders might be associated with elevated uric acid levels.

Significant increases in the medium- and long-chain acylcarnitines were observed in HUA athletes who failed to respond to the DAG diet intervention. Using the metabolomics approach, long-chain acylcarnitines were significantly associated with a higher incidence of HUA in community-dwelling individuals aged 50–70 years old (3). Acylcarnitines function to convey fatty acyls across the inner mitochondria membrane to facilitate fatty acid β-oxidation that generates energy, and elevation in circulating acylcarnitines usually reflects incomplete mitochondrial catabolism and compromised β-oxidation (54). DAG diet intervention (90% DAG and 10% TAG for 8 months) was previously shown to upregulate β-oxidation in C57BL/6J mice, with elevated expressions of acyl-CoA oxidase and medium-chain acyl-CoA dehydrogenase (55). Thus, reduced levels of circulating acylcarnitines in responders might be attributed to enhanced β-oxidation induced by an effective DAG diet intervention. In rat hepatocytes, endogenous uric acid levels were also reported to alter in accordance with the capacity of ATP production (56). In the presence of inhibitors of mitochondrial oxidative phosphorylation (eg rotenone, antimycin, and oligomycin), lowered ATP production was associated with elevated uric acid (56). Therefore, uric acid production in mammalian liver is modulated by changes in hepatic mitochondrial ATP production. Aligned with these findings, we observed positive correlations between serum AST and ALT levels with circulating acylcarnitines (Fig. 5), which cumulatively suggest that an ineffective DAG diet intervention that fails to uplift mitochondrial β-oxidation of fatty acids (reflected by increased serum acylcarnitines) increase ATP production might trigger elevated uric acid production in the liver of non-responders. The resultant xanthine oxidase-derived ROS generation that accompanies hepatic uric acid production might exacerbate the cleavage of vinyl ether linkages in plasmalogen phospholipids, resulting in lower circulating PC Os in non-responders. Indeed, alterations in plasmalogen phospholipids had been observed in HUA and gout individuals (57). Taken together, omics data analyses indicated reduced oxidative stress in responders of the DAG diet intervention.

Aside from lowering hepatic uric acid production, DGCA analysis uncovered perturbed coregulation between phospholipids and metabolites of p-Cresol in responders relative to non-responders (Fig. 4). p-Cresol reduces the transport of uric acid from blood to the peritoneal cavity, thereby modulating the intestinal excretion of uric acid (41, 42). Presumably, diminished levels of p-Cresol and its associated metabolites might increase enteric excretion of uric acid in responders compared to non-responders, which serves to further lower serum uric acid levels. Precise mechanisms on how circuiting phospholipids regulate p-Cresol metabolism warrant further investigation, but a preceding study reported that exogenous administration of PCs improved intestinal barrier defense against toxins secreted by *Clostridium difficile* (58), which is amongst the top four gut microbial strains primarily responsible for the production of p-Cresol from tyrosine (59). PCs are also important constituents of the intestinal mucosal barrier (58). Taken together, non-responders with diminished phospholipids and increased p-Cresol metabolites exhibit abated enteric excretion of uric acid, which might be related to altered gut microbial composition and weakened intestinal barrier function. Augmented ammonia recycling and increased renal ammonia excretion were shown to reduce the endogenous flux of nitrogen directed for uric acid formation (60). Accordingly, responders who displayed enhanced ammonia recycling possess greater efficiency of ammonia excretion in the kidneys that protect them from uric acid overproduction. Indeed, chronic HUA was associated with an increased risk of developing kidney disease (61, 62).

Finally, we explored the possible reasons underlying differential responses to DAG diet intervention in the group of young athletes investigated. Of interest, we discovered that baseline triglycerides were significantly lower in responders compared to non-responders for both males and females (Table 1). A previous study on the mechanism of DAG uptake and absorption revealed that lipoprotein lipase (LPL)-mediated hydrolysis is a crucial determinant of DAG assimilation (63). Accordingly, the lower ambient triglyceride levels in the circulation of responders at baseline would expectably present less competition for LPL action, as endogenous and exogenous neutral lipids both compete for a common, saturable removal system capped by LPL activity (64). Lower triglyceride levels at baseline thus facilitate more efficient lipolysis and DAG assimilation into the peripheral tissues of responders compared to non-responders, thereby contributing to the observed differences in response to DAG diet intervention. Therefore, non-responders might benefit from triglyceride-lowering treatment prior to DAG dietary intervention.

In summary, our exploratory study underscores that the effectiveness of DAG diet intervention in young athletes with HUA might be attributed to disparate triglyceride levels that influence the process of LPL-mediated DAG assimilation into peripheral tissues. In responders with lower triglycerides at baseline, the effective assimilation of DAG was associated with increased mitochondrial β-oxidation, coupled with lesser ROS and uric acid production from the purine metabolism pathway. Reduced ROS generation protects circulating plasmalogen phospholipids such as PC Os from oxidative attacks. Metabolite coregulation analysis further suggests that enhanced phospholipid levels in responders were associated with diminished p-Cresol metabolism that might improve intestinal excretion of uric acid. In addition, enhanced ammonia recycling and renal ammonia excretion serve to reduce nitrogen flux into uric acid production, cumulatively contributing to lower serum uric acid levels in responders. Molecular cues derived from combinatorial lipidomics and metabolomics analyses of human sera, however, warrant further mechanistic verification in the murine model of HUA. In addition, it is difficult to control the precise amount of DAG consumption in individual athletes, as well as the nature and intensity of exercise during the intervention period. Precise modulation of these possible confounders may be feasible only in future animal studies. Nonetheless, the risk of developing metabolic diseases increases with elevated levels of uric acids (12, 13, 14, 15). In atherosclerosis, changes in lipoprotein metabolism, especially the low-density lipoprotein and high-density lipoprotein, were associated with elevated uric acid and low-grade inflammation (38). A significantly elevated ratio of uric acid to high-density lipoprotein (UHR) has been observed in patients with metabolic syndrome, and high UHR increases the risk of developing metabolic syndrome (65). Therefore, dietary intervention to modulate serum uric acid levels is necessary and potentially important not just for athletes, but also for improving general public health. Taken together, our results are indicative that successful implementation of DAG diet intervention has the propensity to mitigate excess uric acid levels resulting from hepatic overproduction and impeded enteric excretion.

## Data Availability

The data of this study can be obtained by contacting the corresponding authors upon reasonable request.

## Supplemental data

This article contains supplemental data.

## Conflict of interest

The authors declare that they have no conflicts of interest with the contents of this article.

## Supplementary Data

Supplementary Tables Supplementary Table 1. Serum lipids associated with responders. A linear model controlling for age, gender, baseline uric acid levels, and the number of dining days were constructed to interrogate the changes in serum lipids between responders and nonresponders. P values were determined by Limma analysis. AveExpr, average log2-expression; adj P.Val, adjusted p-value; CI.L, left limit of confidence interval of log FC; CI.R, right limit of confidence interval of log FC; Log FC, log2-fold-change; t, t-statistic; B, log-odds that the gene is differentially expressed. Supplementary Table 2. Serum metabolites associated with responders. A linear model controlling for age, gender, baseline uric acid levels, and the number of dining days were constructed to interrogate the changes in serum metabolites between responders and nonresponders. P values were determined by Limma analysis. adj P.Val, adjusted p-value; CI.L, left limit of confidence interval of log FC; CI.R, right limit of confidence interval of log FC; AveExpr, average log2-expression; Log FC, log2-fold-change; t, t-statistic; B, log-odds that the gene is differentially expressed.

Supplementary Figures Supplementary Fig. 1. Quality control across MS runs. Left panel: Principal component analysis plots indicated that quality control samples were clustered in close proximity, thus exhibiting that the signals were stable and high data quality during the MS analyses. Right panel: Strong correlations of the quantitated metabolites in the 4 QC samples inserted at the frequency of 1 QC sample per ten biological samples across the MS runs indicated high consistency throughout the MS analyses for (A) lipidomics and (B) metabolites. Supplementary Fig. 2. Boxplot of the top 20 statistically significant lipids. P values were determined by Limma analysis. Majority of the top 20 lipids were PC Os which were significantly elevated in the responders. Supplementary Fig. 3. Boxplot of the remaining 12 of the top 20 statistically significant metabolites. P values were determined by Limma analysis. DHEA, dehydroepiandrosterone. Supplementary Fig. 4. DAG and TAG associated with clinical characteristics. Correlation was calculated using spearman correlation and p-value <0.05 were displayed as dots. Size of the dots indicated magnitude of the -log10 (pvalue). Red denoted positive correlation and blue denoted negative correlation. BMI, body mass index. Supplementary Fig. 5. Schematic summary on potential interorgan crosstalk implicated in the modulation of systemic uric acid levels in responders of DAG diet intervention. Purine metabolism pathway begins with ribose-5-phosphate and ATP that eventually leads to the production of xanthine and uric acid, with concomitant generation of reactive oxygen species (ROS). Lower uric acid level in responders indicates attenuated uric acid production and diminished ROS generation via purine metabolism pathway, possibly contributing to a higher level of circulating PC Os in responders relative to nonresponders. Lower serum acylcarnitines in responders suggest enhanced mitochondria beta-oxidation possibly ascribed to enhanced DAG uptake. Strong correlations between serum acylcarnitines with ALT and AST implied that changes in circulating acylcarnitines were largely ascribed to hepatic mitochondrial activity. Uric acid production occurs in the liver and is inversely correlated with hepatic ATP production. Pertaining to the intestines, increased circulating phospholipids were associated with decreased p-Cresol metabolites that consequently augment enteric excretion of uric acid in responders. In addition, increased ammonia recycling in the kidneys of responders might serve to divert nitrogen resources away from uric acid overproduction. Metabolites shown in pink rectangles were significantly reduced in responders and lipid shown in yellow rectangle/oval was significantly increased in responders. Dashed arrow denotes potential pathway implications. Blue arrow denotes reduction in ROS-associated effects observed in responders. XO, xanthine oxidase; XDH, xanthine dehydrogenase; ROS, reactive oxygen species; PC O, plasmalogen phosphatidylcholine; ATP, adenosine triphosphate; ALT, alanine transaminase; AST, aspartate aminotransferase.
