# Supplementary material for: Lipidomics and metabolomics investigation into the effect of DAG dietary intervention on hyperuricemia in athletes
Source: J Lipid Res. 2024 Jul 25;65(9):100605. doi: 10.1016/j.jlr.2024.100605 (PMC11416290; doi:10.1016/j.jlr.2024.100605)

Supplementary Fig. 1

A

Lipidomics quality control

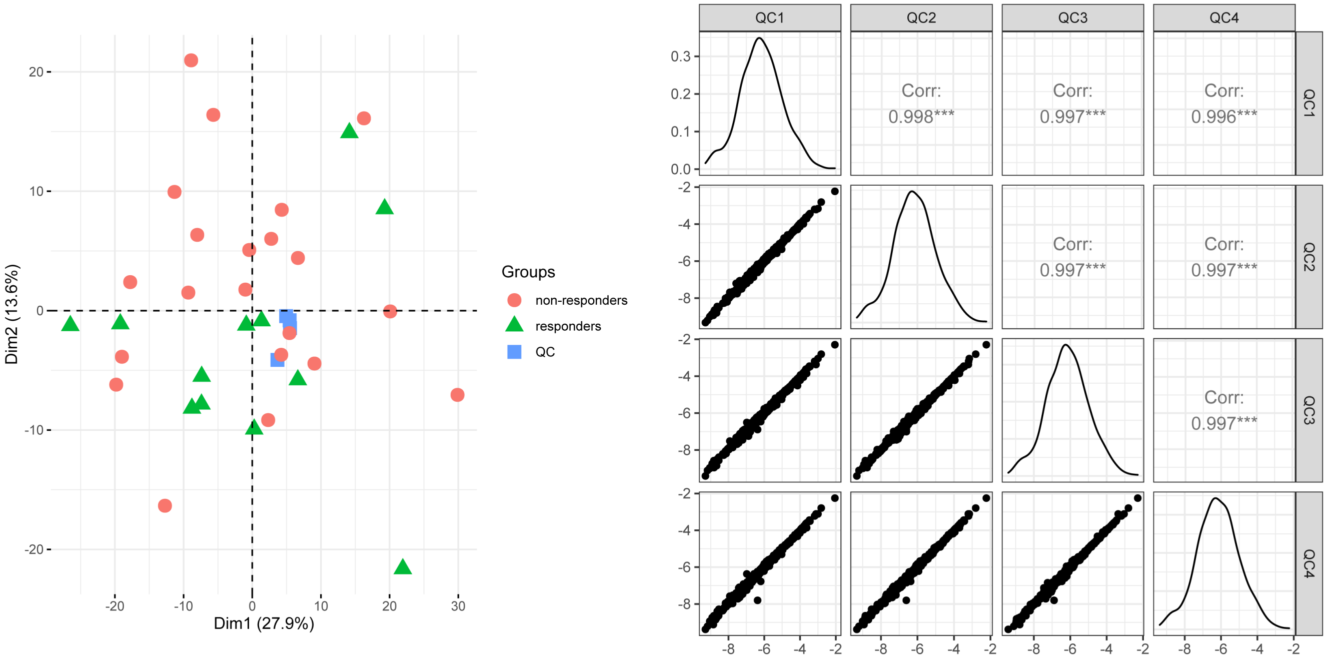

B

Metabolomics quality control

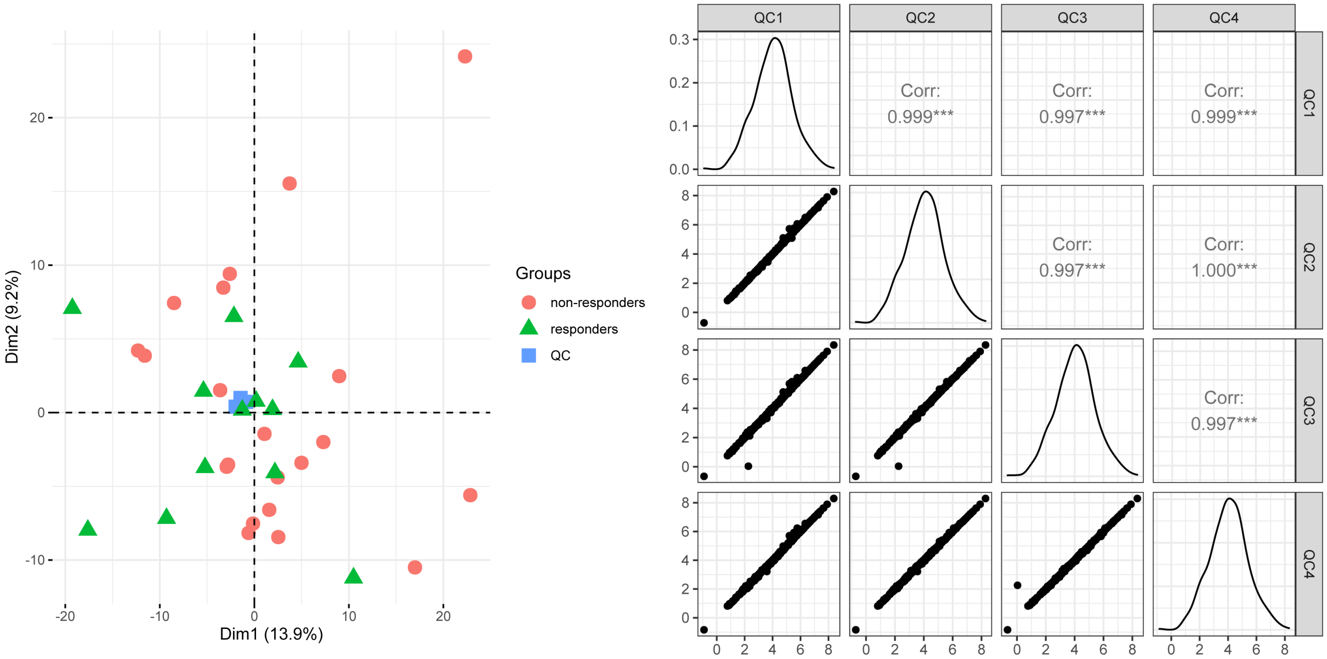

Supplementary Fig. 2

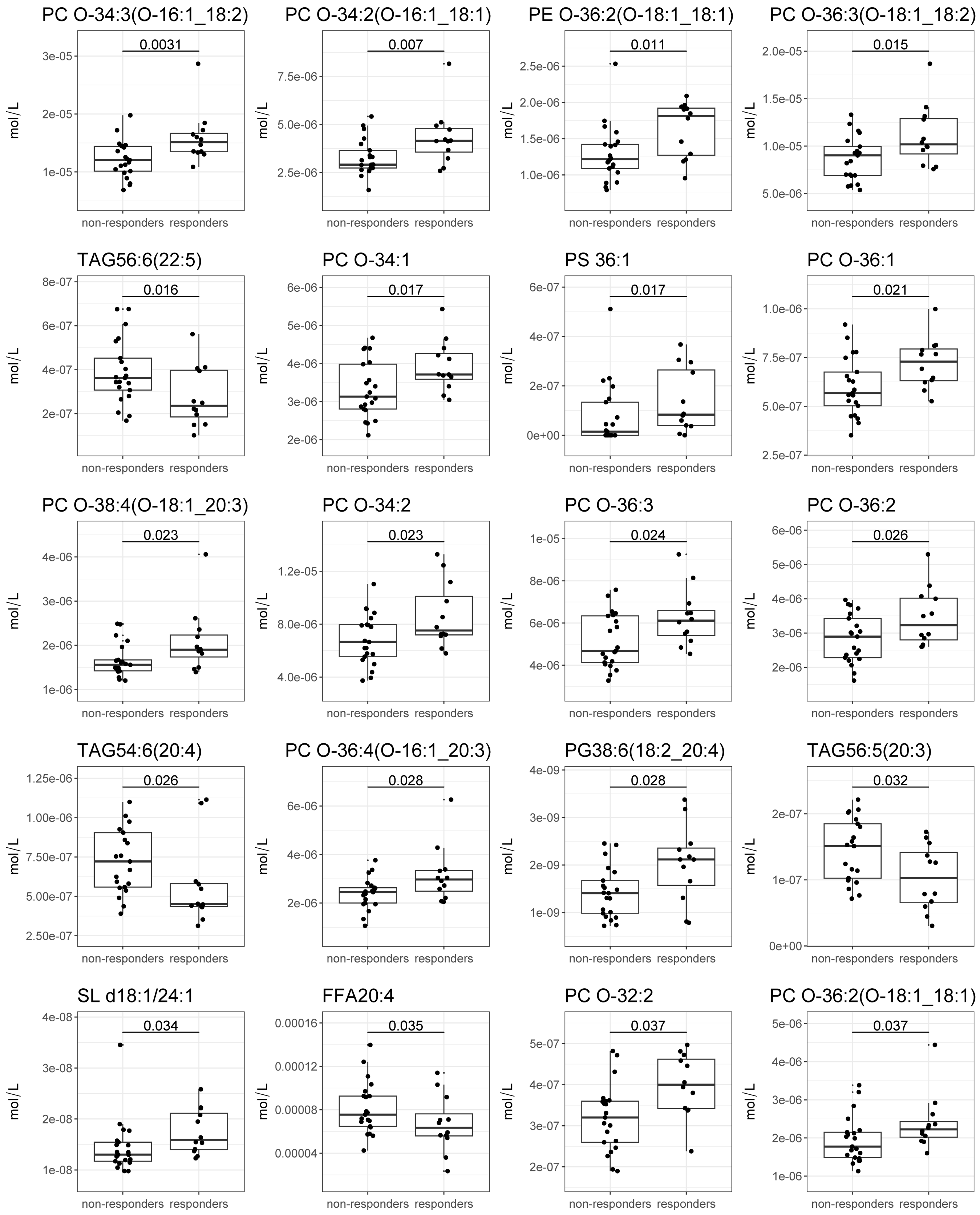

Supplementary Fig. 3

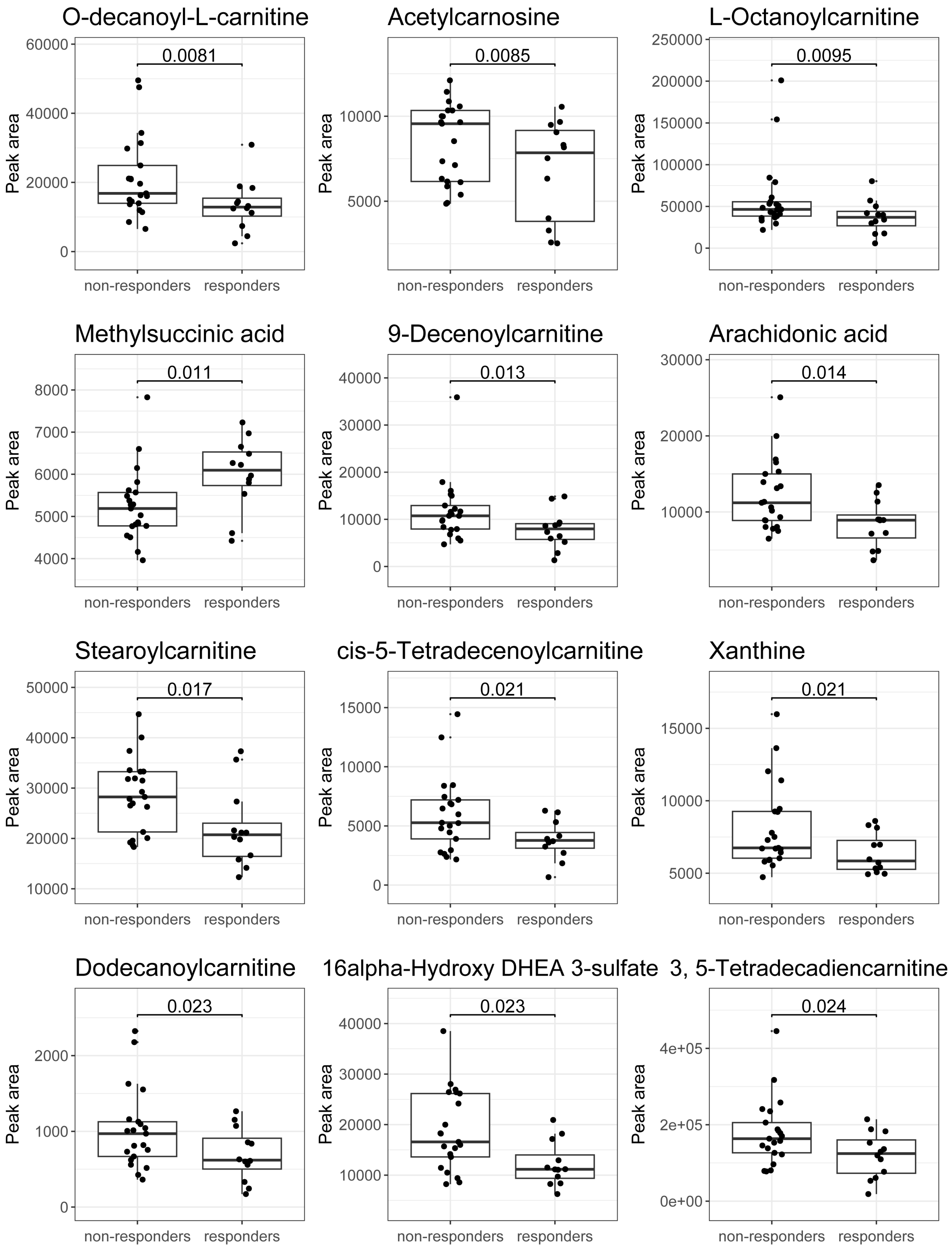

Supplementary Fig. 4

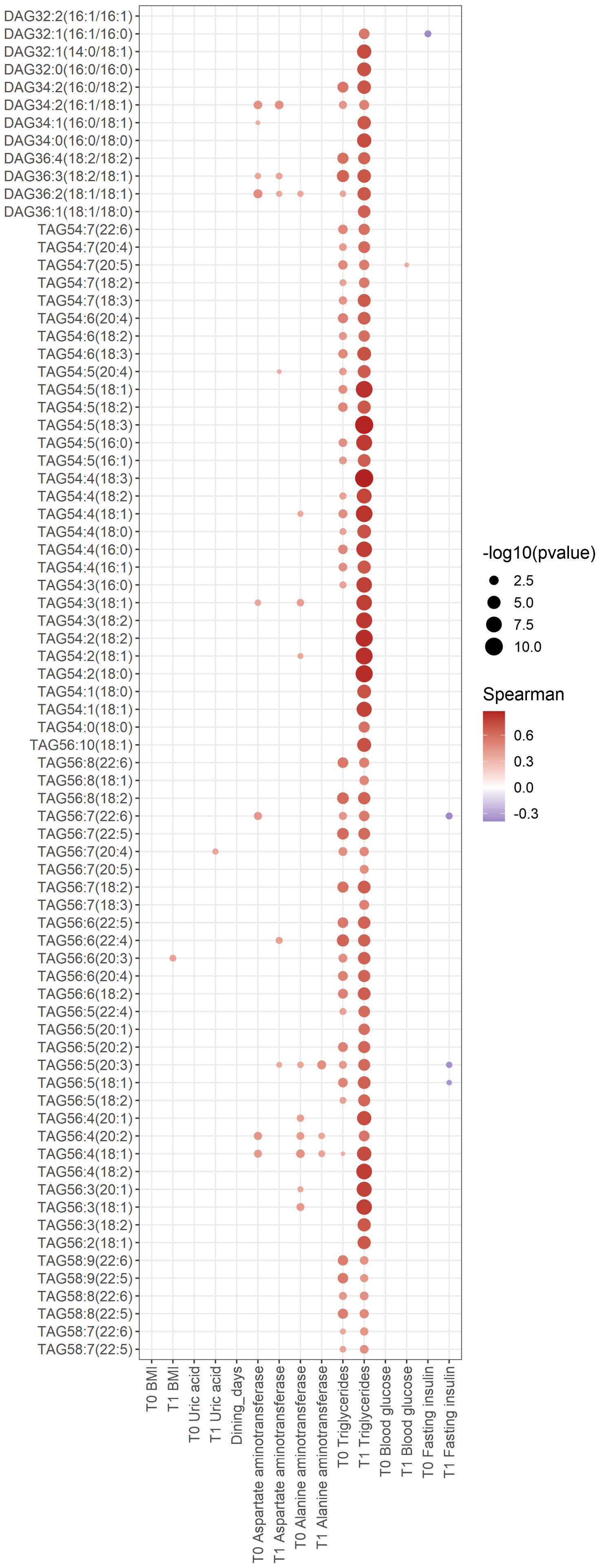

Supplementary Fig. 5

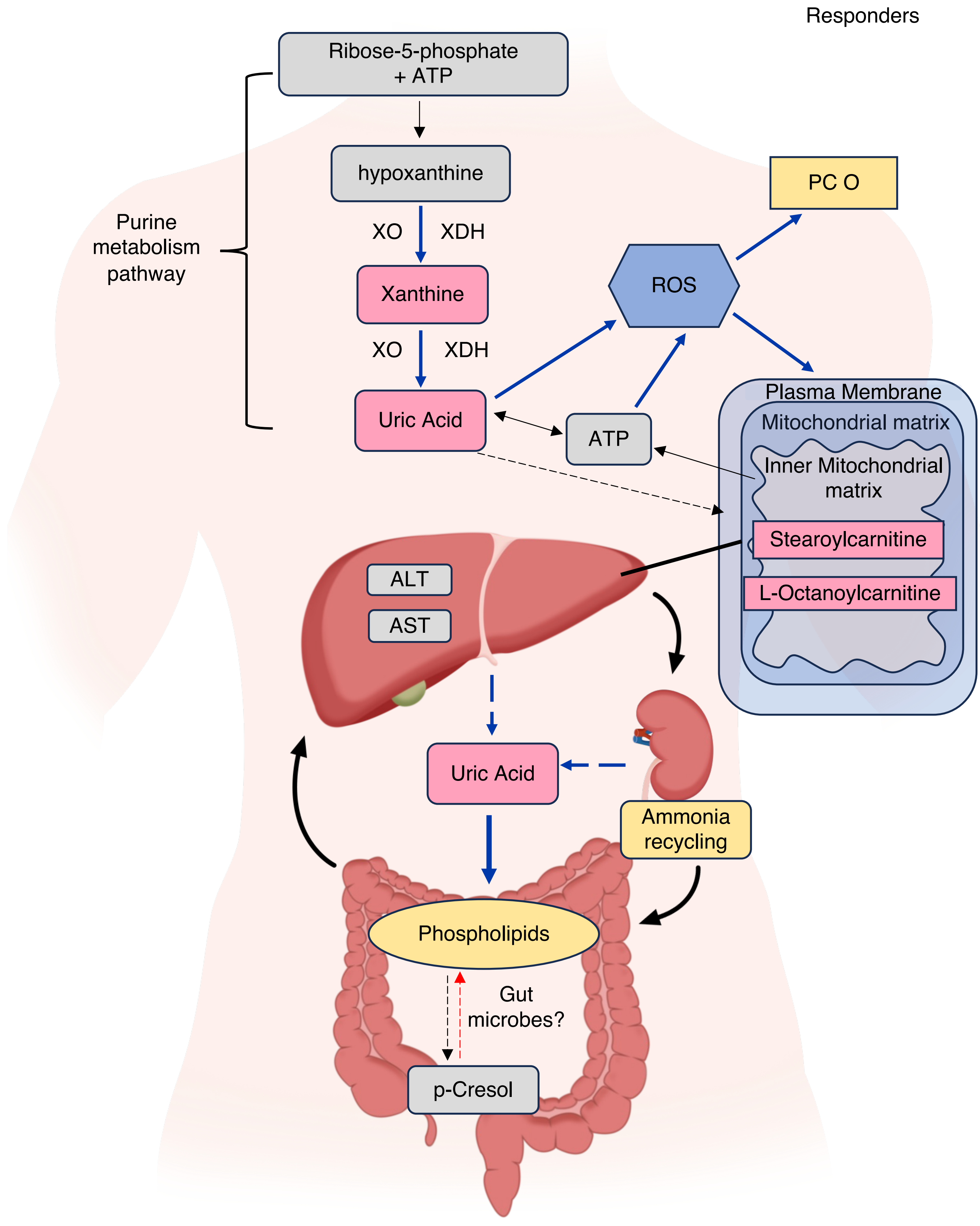

Supplement: Supplementary Figures — Supplementary Fig. 1. Quality control across MS runs. Left panel: Principal component analysis plots indicated that quality control samples were clustered in close proximity, thus exhibiting that the signals were stable and high data quality during the MS analyses. Right panel: Strong correlations of the quantitated metabolites in the 4 QC samples inserted at the frequency of 1 QC sample per ten biological samples across the MS runs indicated high consistency throughout the MS analyses for (A) lipidomics and (B) metabolites. Supplementary Fig. 2. Boxplot of the top 20 statistically significant lipids. P values were determined by Limma analysis. Majority of the top 20 lipids were PC Os which were significantly elevated in the responders. Supplementary Fig. 3. Boxplot of the remaining 12 of the top 20 statistically significant metabolites. P values were determined by Limma analysis. DHEA, dehydroepiandrosterone. Supplementary Fig. 4. DAG and TAG associated with clinical characteristics. Correlation was calculated using spearman correlation and p-value <0.05 were displayed as dots. Size of the dots indicated magnitude of the -log10 (pvalue). Red denoted positive correlation and blue denoted negative correlation. BMI, body mass index. Supplementary Fig. 5. Schematic summary on potential interorgan crosstalk implicated in the modulation of systemic uric acid levels in responders of DAG diet intervention. Purine metabolism pathway begins with ribose-5-phosphate and ATP that eventually leads to the production of xanthine and uric acid, with concomitant generation of reactive oxygen species (ROS). Lower uric acid level in responders indicates attenuated uric acid production and diminished ROS generation via purine metabolism pathway, possibly contributing to a higher level of circulating PC Os in responders relative to nonresponders. Lower serum acylcarnitines in responders suggest enhanced mitochondria beta-oxidation possibly ascribed to enhanced DAG uptake. Strong correla [file mmc2.pdf]
